# Supplementary material for: Structural dynamics of mitochondrial ATP synthase in Chlamydomonas reinhardtii revealed by in situ CryoET
Source: bioRxiv. 2025 Sep 16:2025.09.10.674987. Preprint. [Version 1] doi: 10.1101/2025.09.10.674987 (PMC12458427; doi:10.1101/2025.09.10.674987)
Supplement: Supplement 4 [file NIHPP2025.09.10.674987v1-supplement-4.pdf]

bioRxiv preprint doi: https://doi.org/10.1101/2025.09.10.674987; this version posted September 16, 2025. The copyright holder for this preprint (which was not certified by peer review) is the author/funder, who has granted bioRxiv a license to display the preprint in perpetuity. It is made available under aCC-BY 4.0 International license.

Supplementary materials

Subunits in approximate structural order	NCBI accession number of best match*					BLASTp percent identity *P→C*	Compartment of residence	
	Polytomella sp.		*C. reinhardtii*					
	Protein				Nucleotide		Membrane	Matrix
		AA#		AA#				
Alpha (α)	6RD4_T	562	XP_042927613.1	569	XM_043059979.1	83.33		Yes
Beta (β)	6RD4_X	574	XP_001691632.1	574	XM_001691580.2	90.50		Yes
OSCP	6RD4_P	229	XP_001695985.1	233	XM_001695933.2	62.28		Yes
ASA2	6RD4_2	441	XP_001696742.1	494	XM_001696690.2	44.99		Yes
ASA4	6RD4_4	294	XP_001693576.1	325	XM_001693524.2	50.17		Yes
ASA7	6RD4_7	190	XP_001696750.1	207	XM_001696698.2	50.00		Yes
ASA1	6RD4_1	618	XP_001692395.1	575	XM_001692343.2	54.35		Yes
ASA5	6RD4_5	123	XP_001697115.1	123	XM_001697063.2	65.57	Yes	Yes
ASA3	6RD4_3	325	XP_001700079.1	366	XM_001700027.2	52.01	Yes	Yes
ASA6	6RD4_6	151	XP_001701878.2	150	XM_001701826.2	57.97	Yes	Yes
ASA8	6RD4_8	89	XP_001695222.1	89	XM_001695170.2	78.65	Yes	
ASA9	6RD4_9	97	XP_042920916.1	111	XM_043065751.1	63.64	Yes	
ASA10	6RD4_0	82	XP_042918301.1	95	XM_043068065.1	66.22	Yes	
a-subunit (or 6)	6RD4_M	327	XP_001689492.1	340	XM_001689440.2	66.87	Yes	
c-subunit**	6RD4_A	127	XP_001701500.1	157	XM_001701448.2	75.86	Yes	
			XP_001701531.1	159	XM_001701479.2	65.56		
Delta (δ)	6RD4_R	199	XP_042919463.1	202	XM_043067466.1	68.69		Yes
Epsilon (ε)	6RD4_Q	74	XP_001702609.1	75	XM_001702557.2	63.38		Yes
Gamma (γ)***	6RD4_S	317	XP_042916388.1	324	XM_043070524.1	71.52		Yes
			XP_042916387.1	343	XM_043070523.1	67.54		

Table S1. ATP synthase protein components of Chlorophycean algae.

bioRxiv preprint doi: https://doi.org/10.1101/2025.09.10.674987; this version posted September 16, 2025. The copyright holder for this preprint (which was not certified by peer review) is the author/funder, who has granted bioRxiv a license to display the preprint in perpetuity. It is made available under aCC-BY 4.0 International license.

*Protein and nucleotide records for *C. reinhardtii* were chosen by performing tBLASTn on the protein sequences described in [7], picking the best nucleotide match(es)—and their respective protein records—within *C. reinhardtii* [41].

**It's not clear what combination of the known *c*-subunit mRNAs correspond to the protein that ends up in mitochondrial ATP synthase (the absence of second transcripts for others is taken here to mean that there is only one version). Using protein localization prediction algorithms TargetP 2.0 [42] and MULocDeep [43] respectively, predictions for XP_001701500.1 had more favorable probabilities for mitochondrial localization over chloroplastic (mt: 57.3%, 55.4%; ch: 25.4%, 13.9%) than XP_001701531.1 (mt: 25.0%, 53.7%; ch: 61.6%, 31.4%). Thus, they are accordingly ranked in Table S1. The accuracy of localization prediction becomes more biased for proteins from species whose evolutionary distance from the data used in model training is larger, a problem more severe for algae in TargetP [44]. However, for the purposes of the *C. reinhardtii* ATP synthase protein assembly this distinction is not relevant, as the C-terminus of *c*-subunit included in the c-ring is identical for both.

***The participation of the two versions of gamma is also unclear, but they are not distinguishable by signal sequence because they differ only by an inserted 19 AA on the part of XP_042916387.1. When constructing the *C. reinhardtii* protein-fitted ATP synthase model using XP_042916387.1, it caused clashes with delta and epsilon, and is thus ranked below XP_042916388.1.

bioRxiv preprint doi: https://doi.org/10.1101/2025.09.10.674987; this version posted September 16, 2025. The copyright holder for this preprint (which was not certified by peer review) is the author/funder, who has granted bioRxiv a license to display the preprint in perpetuity. It is made available under aCC-BY 4.0 International license.

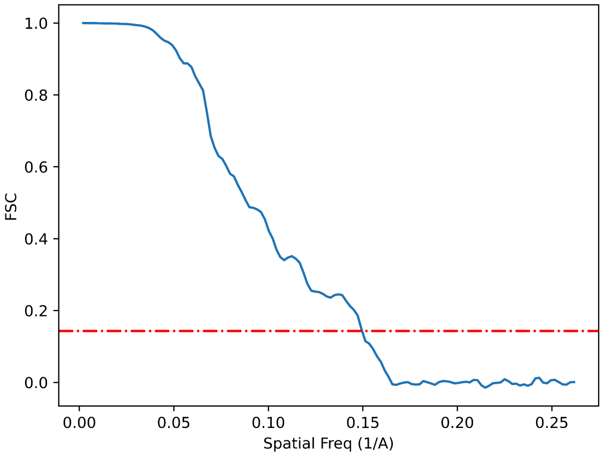

Figure S1. FSC resolution curve. At an FSC of 0.143, the resolution of the map in Figure 1 is 6.67Å.

bioRxiv preprint doi: https://doi.org/10.1101/2025.09.10.674987; this version posted September 16, 2025. The copyright holder for this preprint (which was not certified by peer review) is the author/funder, who has granted bioRxiv a license to display the preprint in perpetuity. It is made available under aCC-BY 4.0 International license.

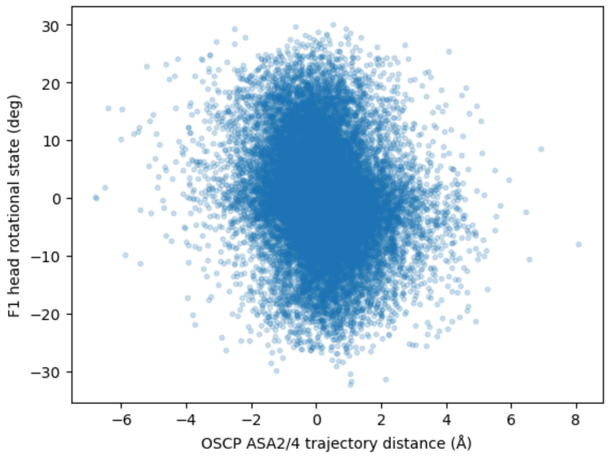

Figure S2. Particles' F1 head rotational state against their DNN-assigned OSCP trajectory distance. A non-random relationship between the upper peripheral stalk trajectory distance and F1 head rotational state is shown, indicating the existence of correlated movement.

bioRxiv preprint doi: https://doi.org/10.1101/2025.09.10.674987; this version posted September 16, 2025. The copyright holder for this preprint (which was not certified by peer review) is the author/funder, who has granted bioRxiv a license to display the preprint in perpetuity. It is made available under aCC-BY 4.0 International license.

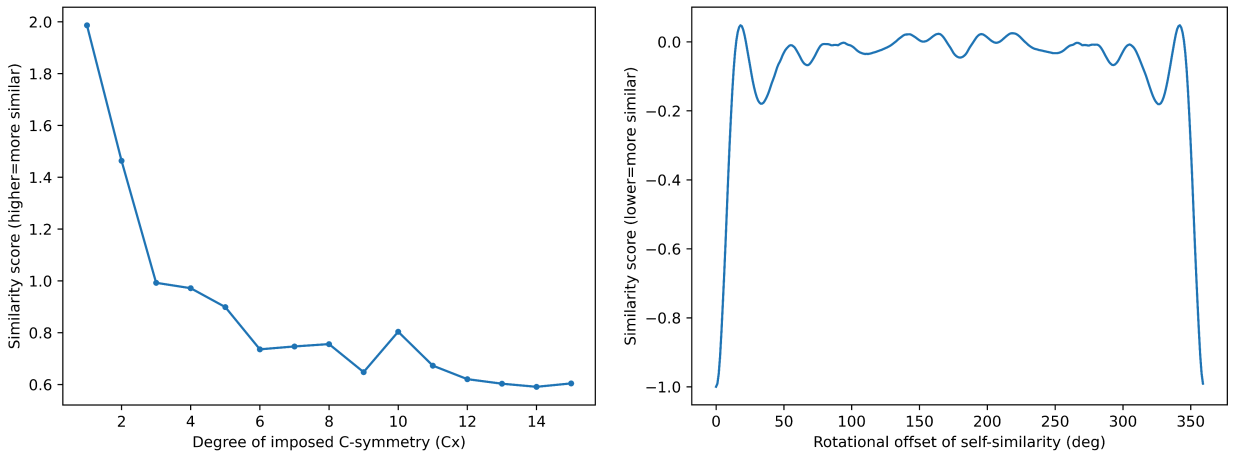

Figure S3. Similarity scores for imposed symmetry and rotational similarity performed on the *c*-ring volume. (a) Several C symmetries were imposed on the model's *c*-ring and similarity to the original was calculated, where a higher score indicates more similarity. Note the peak at C10. (b) The *c*-ring volume was self-imposed and a similarity score calculated for each degree of rotation around the C symmetry axis, a lower score indicating more similarity. Note the 10 score minima.

bioRxiv preprint doi: https://doi.org/10.1101/2025.09.10.674987; this version posted September 16, 2025. The copyright holder for this preprint (which was not certified by peer review) is the author/funder, who has granted bioRxiv a license to display the preprint in perpetuity. It is made available under aCC-BY 4.0 International license.

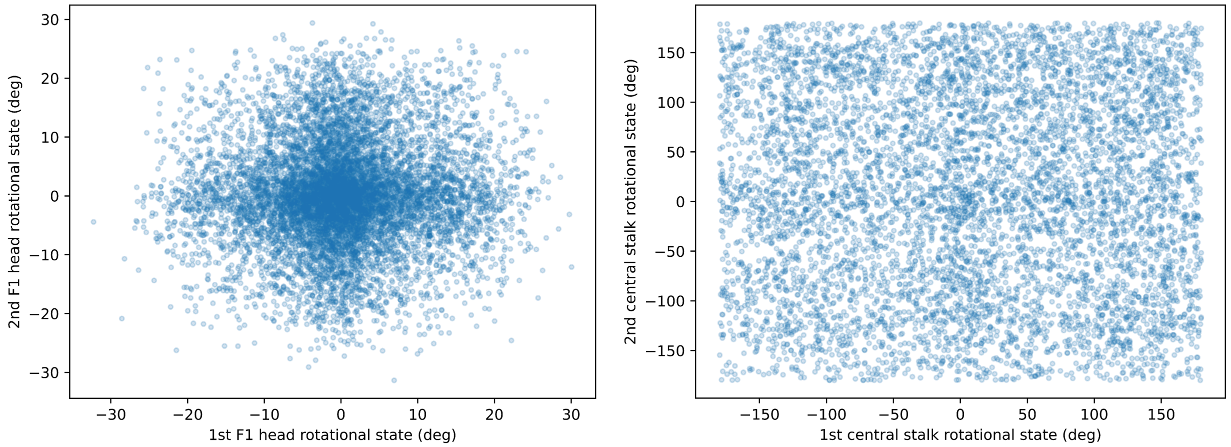

Figure S4. Correlations of intra-dimer F1 heads and central stalks. Each point represents a dimer particle with (a) F1 head and (b) central stalk rotational states assigned for each monomer.

bioRxiv preprint doi: https://doi.org/10.1101/2025.09.10.674987; this version posted September 16, 2025. The copyright holder for this preprint (which was not certified by peer review) is the author/funder, who has granted bioRxiv a license to display the preprint in perpetuity. It is made available under aCC-BY 4.0 International license.

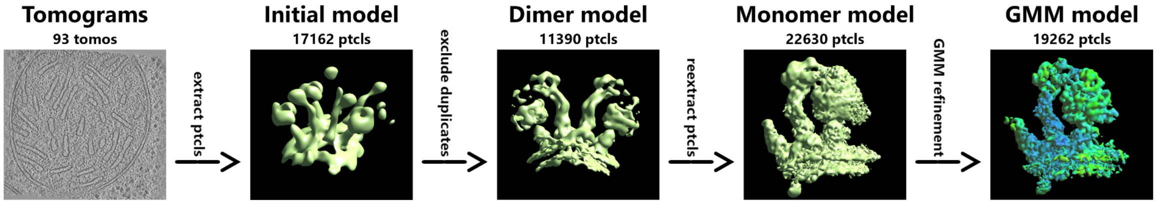

Figure S5. Subtomogram refinement process workflow.
